# Urine Afamin as a biomarker of lupus nephritis

**DOI:** 10.3389/fimmu.2025.1696288

**Published:** 2025-11-28

**Authors:** Shiyu Lin, Mengru Du, Jie Wang, Peng Lai, Genhong Yao, Weiwei Chen, Xue Xu, Lingyun Sun

**Affiliations:** Department of Rheumatology and Immunology, Nanjing Drum Tower Hospital, The Affiliated Hospital of Nanjing University Medical School, Nanjing, China

**Keywords:** systemic lupus erythematosus, lupus nephritis, Afamin, rSLEDAI, SLEDAI-2K

## Abstract

**Objective:**

The association between various biomarkers and the risk of lupus nephritis (LN) has been extensively investigated. However, a dearth of readily available, specific, and sensitive diagnostic biomarkers for LN remains. This study aimed to examine the levels of Afamin in the urine and plasma of patients with systemic lupus erythematosus (SLE) and to evaluate its potential utility for diagnosing LN.

**Methods:**

This study enrolled 31 LN–SLE patients, 31 non-LN–SLE patients, and 27 healthy controls (HC). First, tandem mass tag (TMT)–based proteomic screening of urine and plasma (*n* = 15 per group) identified Afamin as a significantly upregulated protein in LN. Subsequently, ELISA validation confirmed that both uAfamin and pAfamin levels were markedly higher in the LN group. The diagnostic performance of uAfamin for active LN was further evaluated using receiver operating characteristic (ROC) curve analysis.

**Results:**

Levels of both urinary and plasma Afamin were significantly elevated in patients with LN–SLE compared to those with non-LN–SLE. Moreover, uAfamin and pAfamin levels demonstrated robust correlations with multiple clinical indicators of disease activity, including 24h urinary total protein (24hUTP), creatinine, urea, hematuria, pyuria, SLEDAI, and renal SLEDAI. Multivariable linear regression analysis established uAfamin as an independent determinant of renal SLEDAI scores. Consistent with this, ROC curve analysis confirmed that uAfamin possesses a strong capacity to diagnose LN.

**Conclusion:**

The uAfamin level serves as a biomarker for LN, aiding in monitoring disease progression and suggesting a new avenue for personalized treatment strategies.

## Introduction

Systemic lupus erythematosus (SLE) is a complex systemic autoimmune disorder (1) characterized by immunological dysregulation, multi-organ damage, and a fluctuating course of flares and remissions that can result in irreversible injury (2). Lupus nephritis (LN), a common and serious renal complication, is a major predictor of adverse outcomes, with reported 5-year survival rates ranging from 46% to 95% despite treatment (3). Although early diagnosis and timely intervention are crucial for improving long-term survival (4), the often insidious onset and variable clinical course of LN complicate effective early detection and monitoring (5, 6).

Several conventional biomarkers are currently applied in the clinical assessment of LN disease activity (7), including proteinuria, the urine protein-to-creatinine ratio, creatinine clearance, anti-double stranded DNA antibodies (anti-dsDNA), and complement component levels. However, the relationship between these markers and LN is not entirely reliable, and their effectiveness in indicating disease activity and forecasting outcomes is still a matter of debate (5). These markers exhibit limitations in both sensitivity and specificity when it comes to distinguishing between renal activity and damage in LN. Notably, significant renal damage may occur prior to any observable impairment in renal function, which is typically identified through laboratory parameters (8).

Therefore, there is a clear need for reliable, accessible biomarkers capable of diagnosing LN flares prior to the manifestation of conventional clinical thresholds, such as significant proteinuria, renal dysfunction, or active urine sediment (9, 10). A biomarker suitable for serial monitoring would allow for the prompt initiation and adjustment of therapy during active or relapsing disease. In the long term, it could also aid in shortening the course of immunosuppressive treatment by enabling earlier confirmation of renal remission (11).

Urinary biomarkers are generally considered more promising than plasma biomarkers owing to their non-invasive collection and their capacity to directly mirror the renal status, as they often represent specific products derived from localized inflammatory activity within the kidney (12). In this study, a comparative proteomic analysis of urine and plasma samples from healthy controls (HC), non-LN–SLE, and LN–SLE patients was conducted using tandem mass tag (TMT) profiling. This approach identified significantly elevated levels of Afamin in LN–SLE patients.

Afamin, a glycoprotein and the fourth member of the albumin superfamily, is primarily produced in the liver and is known to bind and transport vitamin E. Although the liver is its main site of synthesis, afamin is also expressed at lower levels in other tissues, including the brain, kidneys, testes, and ovaries. The detection of Afamin mRNA in the kidney suggests a potential (www.proteinatlas.org/ENSG00000079557-AFM), though not yet fully elucidated, role in renal function.

Previous studies have highlighted the relevance of urine Afamin (uAfamin) with various kidney disorders. Mirela Sedic et al. identified uAfamin as a candidate biomarker for primary membranous nephropathy (PMN) (13). Furthermore, elevated uAfamin levels have been reported in several other renal conditions, including diabetic nephropathy (14), focal segmental glomerulosclerosis (15), IgA nephropathy (IgAN) (16, 17), and pediatric idiopathic nephrotic syndrome (18). Notably, it has been proposed that uAfamin may reflect glomerular barrier integrity rather than filtration capacity (19), suggesting its potential utility as a novel biomarker for active LN (20).

Building on these findings, this study aimed to evaluate the clinical utility of Afamin as a biomarker for distinguishing SLE patients with and without LN and to elucidate its correlation with disease activity.

## Methods

### Patients

This study enrolled 62 patients diagnosed with SLE according to the 2019 European League Against Rheumatism (EULAR)/American College of Rheumatology (ACR) classification criteria (21), alongside 27 age- and sex-matched HC. All participants were recruited from Nanjing Drum Tower Hospital. The SLE cohort was stratified into two groups: a non-LN–SLE group (*n* = 31), comprising patients without significant renal involvement (as indicated by normal serum creatinine and urine sediment), and an LN–SLE group (*n* = 31), which included patients with biopsy-proven and/or clinically significant renal manifestations (e.g., proteinuria and/or elevated serum creatinine).

Individuals with comorbidities that could potentially confound renal assessment or urinary biomarkers were excluded. These conditions included hypertension (unrelated to LN), diabetes mellitus, urinary tract infections, urolithiasis, other urological disorders, acute renal failure, and dehydration.

The study protocol was approved by the Ethics Committee of Nanjing Drum Tower Hospital (Approval No. 2023-639-01) and conducted in accordance with the principles of the Declaration of Helsinki. Written informed consent was obtained from all participants.

### Clinical assessment

Comprehensive medical histories, including demographic data, clinical manifestations, physical examination findings, and current treatments, were obtained from medical records and case managers. Disease activity was assessed using the SLE Disease Activity Index–2K (SLEDAI-2K), which has a theoretical range of 0–105, with a score of 0 indicating no disease activity (22). Renal involvement was evaluated using the renal SLEDAI (rSLEDAI), with scores ranging from 0 (no renal disease activity) to a maximum of 16 (23). According to criteria from Schwartz et al. (6) and Zhu et al. (24), an rSLEDAI score ≥ 4 was defined as active LN (25). The LN–SLE group was further categorized into two subgroups based on the severity of renal disease: patients with severe active LN (*n* = 12, rSLEDAI score ≥12) and those with mild active LN (*n* = 4, 12 > rSLEDAI score ≥4) (22).

### Laboratory tests

Laboratory investigations included blood routine examination, erythrocyte sedimentation rate (ESR), urine analysis, serum creatinine, blood urea nitrogen (BUN), serum albumin, serum complement factors 3 (C3) and serum complement factors 4 (C4), antinuclear antibody (ANA), anti-dsDNA, and 24h urinary total protein quantification (24hUTP), which were performed for all patients. Abdominal ultrasound and renal biopsy were carried out for patients with LN. An ultrasound apparatus (grayscale) manufactured by Philps (Japan) with transducer frequency (2.5–5 MHz) and with a convex probe was used. Renal pathology was classified based on the revised International Society of Nephrology and Renal Pathology Society (ISN/RPS) 2018 classification (25) and could be grouped by activity index (AI) (low, 0–5; mild, 6–11; high, 12–24).

### Tandem mass tags proteomic analysis

This discovery-phase proteomic study analyzed serum and urine samples from 15 LN–SLE patients, 15 non-LN–SLE patients, and 15 HC. Sample preparation and protein digestion were performed according to the manufacturer’s Thermo Fisher Scientific (Waltham, MA, USA) protocols. Equal amounts of protein from each sample were subjected to TMT labeling. After labeling, the samples were combined in equal proportions, desalted using Sep-Pak C18 cartridges, and vacuum dried. The dried samples were then fractionated using a high-pH reverse-phase chromatography method. The resulting fractions were either stored at −80 °C or directly analyzed using a Q Exactive Plus liquid chromatography–mass spectrometry (LC-MS) system (Thermo Fisher Scientific).

The raw mass spectrometry data were processed with MaxQuant software (version 1.6.6) using the Andromeda search engine. The database search was filtered at a 1% false discovery rate (FDR) at both the protein and peptide levels. Proteins identified as reverse hits, potential contaminants, or those supported by only a single modified peptide were excluded from further analysis. Subsequently, functional enrichment analysis of the differentially expressed proteins was performed using hypergeometric tests to identify statistically overrepresented biological categories.

### Enzyme-linked immunosorbent assay

To validate the findings from the proteomic discovery phase, the levels of differentially expressed proteins were measured using enzyme-linked immunosorbent assay (ELISA). Based on the proteomic profiling results, Afamin was selected for subsequent validation in both serum and urine. Its concentrations were quantified using commercial ELISA kits (CUSABIO) according to the manufacturer’s protocols. All samples were assayed in duplicate under standardized conditions to ensure the consistency and accuracy of the measurements.

### Statistical analysis

Data were collected and analyzed using the Statistical Package for the Social Sciences (SPSS, version 17). Continuous data with a normal distribution are presented as mean ± standard deviation (SD), while data with a non-normal distribution are expressed as median (interquartile range). For comparisons between two groups, the Fisher’s least significant difference (LSD) t-test or the Mann–Whitney *U* test was applied, depending on whether the data followed a normal distribution. Correlations were assessed using Spearman’s rank correlation coefficient. The influence of laboratory parameters on the renal SLEDAI (rSLEDAI) was evaluated with linear regression analysis. The diagnostic performance of urinary Afamin (uAfamin) in discriminating between SLE patients with and without LN was assessed by calculating the area under the curve (AUC) of nonparametric receiver operating characteristic (ROC) curves. A two-sided *P*-value of less than 0.05 was considered statistically significant.

## Results

### Patient characteristics

This study enrolled a total of 62 SLE patients, who were categorized into a non-LN–SLE group (*n* = 31) and an LN–SLE group (*n* = 31). From the LN–SLE cohort, 16 patients (excluding the 15 used in the TMT assay) were further stratified by renal disease activity into mild active LN (*n* = 4) and moderate-to-severe active LN (*n* = 12) based on their rSLEDAI scores.

Significant differences in clinical and laboratory parameters were observed between the LN–SLE and non-LN–SLE groups. The LN–SLE group had significantly higher median rSLEDAI scores (13 [IQR 8–16] *vs*. 0 [IQR 0–3], *P* < 0.001), SLEDAI scores (20.38 ± 5.48 *vs*. 6.75 ± 5.31, *P* < 0.001), serum creatinine (65.06 ± 18.82 μmol/L *vs*. 48.19 ± 14.00 μmol/L, *P* = 0.006), 24hUTP (1.84 [IQR: 0.98–3.81] g/24h *vs*. 0.14 [IQR: 0.069–0.15] g/24h, *P* = 0.01), and urinary sediment markers (pyuria and hematuria, both *P* < 0.001). In contrast, the LN–SLE group exhibited lower eGFR (109.69 ± 39.10 *vs*. 144.26 ± 44.91 ml/min/1.73 m², *P* = 0.032), complement C3 levels (0.71 ± 0.31 *vs*. 0.98 ± 0.34 mmol/L, *P* = 0.025), and 25(OH)-VD_3_ levels (9.91 ± 6.41 *vs*. 22.27 ± 9.98 μg/L, *P* < 0.001). Detailed comparisons are presented in **Table 1**. The NIH activity index classifications for the LN–SLE subgroups are summarized in **Table 2**.

**Table 1:** Clinical and laboratory features of the patients.

	LN-SLE n=16	Non-LN SLE n=16	statistic	P value
age(year)	35.31±10.14	40.75±16.83	t=-1.11	0.277
male/female, n (%)	1(6.25)/15(93.75)	3(18.75)/13(81.25)	–	0.600
SLE disease duration(year)	3.5(1.5,11.69)	3.3(1.5,10.3)	z=-0.02	0.970
systolic blood pressure(mmHg)	127(117.25,137)	112(104,120)	z=-2.51	0.011
diastolic blood pressure(mmHg)	82.56±13.71	71.33±20.45	t=1.81	0.807
WBC (10^9/L)	6.05(3.67,7.38)	4.90(4.28,5.75)		
Hb (g/L)	103±16.78	112.38±12.75	t=-2.30	0.029
PLT (10^9/L)	215.5(145.75,231.75)	229(182,272.5)	z=-0.51	0.598
CRP (mg/L)	2.5(2.0,2.85)	2.8(2.4,6.0)	z=-1.94	0.05
ESR (mm/1st h)	34.5(16,57)	34.5(15.25,56)	z=-0.25	0.792
serum albumin(g/L)	29.9(21.83,34.68)	35.7(33.35,40)	z=-3.23	0.02
Scr (μmol/L)	65.06±18.82	48.19±14.00	t=2.88	0.006
urea (mmol/L)	6.55(5.1,9.17)	5.55(3.68,6.35)	t=2.50	0.044
eGFR (ml/min/1.73m^2)	109.69±39.10	144.26±44.91	t=-2.32	0.032
24 hUTP (g/24h)	1.84(0.98,3.81)	0.14(0.069,0.15)	z=-4.82	0.01
pyuria(/HPF)	18.06(8.80,37.42)	1.38(0.46,4.14)	z=-4.34	0.001
hematuria(/HPF)	13.94(1.35,13.9)	0.36(0.18,1.17)	z=-3.65	0.001
C3 (mmol/L)	0.71±0.31	0.98±0.34	t=-2.35	0.025
C4 (mmol/L)	0.08(0.05,0.22)	0.17(0.09,0.35)	z=-1.47	0.136
IgA(g/L)	1.88(1.66,2.46)	2.76(2.08,4.10)	z=-2.13	0.032
IgM(g/L)	0.98(0.62,1,31)	0.86(0.41,1.21)	z=-4.9	0.624
IgG(g/L)	9.05(7.9,12.4)	17.75(14.25,27.80)	z=-3.56	0.001
Anti-dsDNA (IU/ml)	297.92(97.42,708.21)	133.02(59.48,347.15)	z=-1.60	0.105
ANA	1000(320,3200)	1000(320,2100)	z=-0.14	0.874
25(OH)-VD_3_(μg/L)	9.91±6.41	22.27±9.98	t=-4.12	0.001
SLEDAI-2K	20.38±5.48	6.75±5.31	t=7.10	0.001
rSLEDAI	13(8,16)	0(0,3)	z=-4.81	0.001

LN, lupus nephritis; SLE, systemic lupus erythematosus; WBC, white blood cell; Hb, hemoglobin; PLT, platelets; ESR, erythrocyte sedimentation rate; CRP, C reactive protein; Scr, serum creatinine; eGFR, estimated glomerular filtration rate; 24 hUTP, 24-hour urinary total protein quantification; HPF, high power field; C3, complement factor 3; C4, complement factor 4; Ig immunoglobulin; Anti-dsDNA anti-double stranded DNA antibody; ANA, antinuclear antibody; 25(OH)-VD_3_ 25(OH)-Vitamin D3; SLEDAI-2K, SLE disease activity index; rSLEDAI, renal SLE disease activity index.

**Table 2:** NIH Lupus Nephritis Activity Index Score of LN patients.

Mild active LN (n=4)	Severe active LN (n=12)	Activity index (AI), n (%)
4	10	Low AI (87.5)
0	1	Mild AI (6.25)
0	0	High AI
	1	Vague (6.25)

Kidney disease activity was assessed by the renal SLEDAI (rSLEDAI), which ranges from 0 (non-active renal disease) to a maximum score of 16: severe active (n=12,rSLEDAI score≥12)and mild active renal disease (n=4,12>rSLEDAI score≥4).

NIH Lupus Nephritis Activity Index Score was classified into low AI (0-5), mild AI (6-11), high AI (12-24).

### Increased levels of Afamin in LN patients

**Figure 1:**
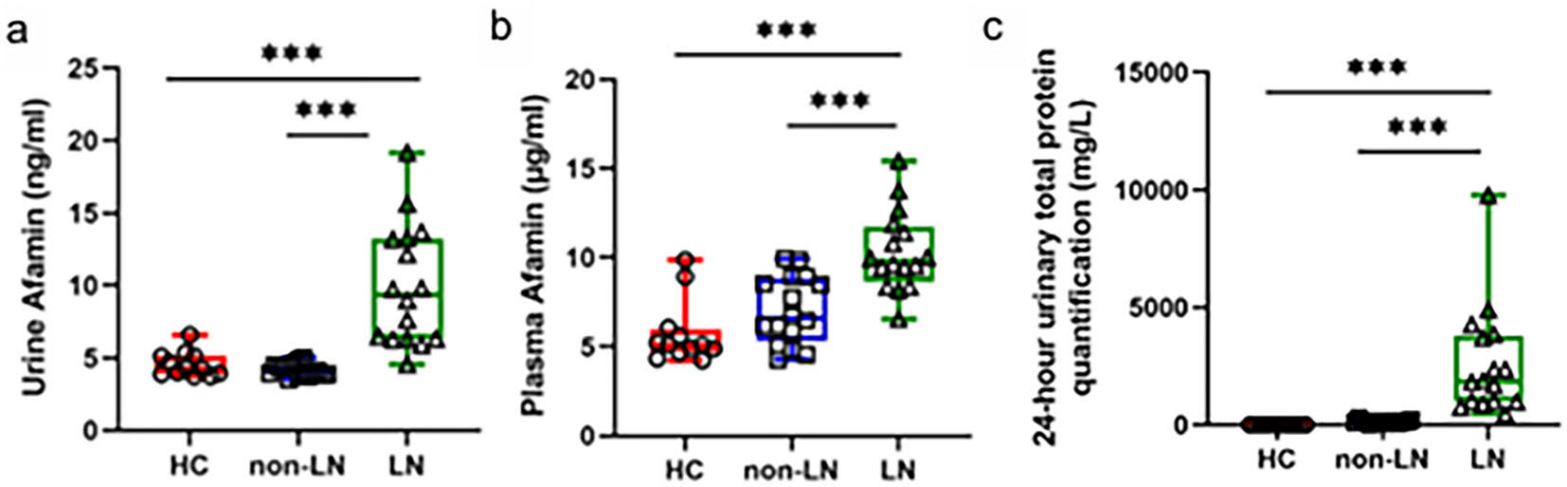
The expression of urine **(A)** and plasma afamin **(B)** by ELISA in healthy controls (n=12), non-LN SLE (n=16) and LN SLE (n=16) groups. **(C)** The levels of 24hUTP in healthy controls (n=12), non-LN SLE (n=16) and LN SLE (n=16) groups. ***P<0.001.

TMT-based proteomic profiling of urine and plasma samples from 15 HC, 15 non-LN–SLE, and 15 LN–SLE patients (demographics in **Supplementary Table S1**) identified 1,477 plasma proteins and 2,440 urine proteins (**Supplementary Figure S1**). Among these, Afaminm was one of the most robustly elevated proteins in both plasma and urine from LN–SLE patients compared to the non-LN–SLE and HC groups. To validate this finding, we quantified Afamin levels in a separate cohort comprising 16 LN–SLE patients, 16 non-LN–SLE patients, and 12 HCs using ELISA. The results confirmed that uAfamin levels were significantly higher in the LN–SLE group (9.93 ± 4.18 ng/ml) than in the non-LN–SLE (4.19 ± 0.42 ng/ml) and HC (4.56 ± 0.86 ng/ml) groups (*P* < 0.001). Similarly, pAfamin levels were also markedly elevated in LN–SLE patients (10.33 ± 2.26 µg/ml) compared to the non-LN–SLE (7.03 ± 1.89 µg/ml) and HC (5.74 ± 1.79 µg/ml) groups (*P* < 0.001) (**Figure 1**).

### Correlation of urine/plasma Afamin with different clinical and laboratory manifestations in LN patients

Correlation analysis showed no significant associations between Afamin levels (in either urine or plasma) and demographic characteristics, including sex and disease duration. Similarly, no significant correlations were found with autoantibody profiles (ANA and anti-dsDNA). In contrast, Afamin levels, particularly urinary Afamin (uAfamin), demonstrated robust positive correlations with key indicators of renal impairment: 24h urinary total protein (24hUTP; *r* = 0.792, *P* = 0.001), serum creatinine (*r* = 0.488, *P* = 0.005), urea (*r* = 0.358, *P* = 0.044), hematuria (*r* = 0.703, *P* = 0.001), and pyuria (r = 0.665, *P* = 0.001). Plasma Afamin (pAfamin) was also correlated with 24hUTP (*r* = 0.530, *P* = 0.002) and pyuria (*r* = 0.497, *P* = 0.004). A negative trend was observed between Afamin levels and estimated glomerular filtration rate (eGFR), although it did not reach statistical significance. Furthermore, we detected significant inverse associations of Afamin with complement C3, serum albumin, hemoglobin, and 25(OH)-Vitamin D3 levels (**Table 3**).

**Table 3:** Relationship between urine/plasm Afamin and laboratory parameters of LN patients.

Variables	uAfamin		pAfamin	
	P Value	*r*	P Value	*r*
ANA	0.943	-0.013	0.253	-0.208
Anti-dsDNA	0.067	0.345	0.375	0.171
C3	0.030	-0.383*	0.093	-0.302
C4	0.153	-0.259	0.377	-0.162
24 hUTP	0.000	0.792**	0.002	0.530**
hematuria	0.000	0.703**	0.061	0.335
pyuria	0.000	0.665**	0.004	0.497**
Scr	0.005	0.488**	0.059	0.338
urea	0.044	0.358*	0.071	0.323
serum albumin	0.033	-0.379*	0.004	-0.496**
eGFR	0.055	-0.343	0.107	-0.290
ESR	0.722	0.065	0.731	0.063
CRP	0.418	-0.148	0.165	-0.252
Hb	0.003	-0.507**	0.006	-0.478**
PLT	0.669	-0.078	0.878	-0.028
LA	0.503	-0.137	0.335	-0.197
D-dimmer	0.104	0.292	0.085	0.310
25(OH)-VD_3_	0.008	-0.468**	0.001	-0.641**
SLEDAI-2K	0.001	0.717**	0.054	0.344
rSLEDAI	0.001	0.796**	0.007	0.471**

LA lupus anticoagulant.

The LN patient data from the ELISA validation cohort (n=16).

* Significant, ** Highly significant.

### Association between urine/plasma Afamin and renal disease activity in LN patients

Urinary Afamin (uAfamin) levels exhibited a robust positive correlation with both the systemic SLEDAI (*r* = 0.717, *P* = 0.001) and the renal-specific rSLEDAI scores (*r* = 0.796, *P* = 0.001). A significant correlation was also observed between plasma Afamin (pAfamin) and rSLEDAI (*r* = 0.471, *P* = 0.007) (**Table 3**).

Univariate linear regression analysis identified uAfamin, 24 hUTP, complement C3, hematuria, and 25(OH)-VD_3_ as variables significantly associated with rSLEDAI. Subsequent multivariate analysis, which adjusted for these potential confounders, confirmed that uAfamin (β = 1.01, *P* = 0.001) remained an independent predictor of renal disease activity. This result underscores the potential of uAfamin as a biomarker for diagnosing the severity of LN (**Table 4**).

**Table 4:** The correlation between laboratory examination and rSLEDAI in patients with LN.

Variables	Simple				Multivariable			
	β	t	P	β (95%CI)	β	t	P	β (95%CI)
pAfamin	1.12	2.84	**0.008**	1.12(0.35 – 1.90)	-0.26	-0.64	0.534	-0.26 (-1.06 - 0.54)
uAfamin	1.06	5.03	**<.001**	1.06 (0.65 - 1.47)	1.01	3.90	**0.001**	1.01 (0.50 - 1.51)
24 hUTP	0.01	4.64	**<.001**	0.01 (0.01 - 0.01)	0.00	1.40	0.181	0.00 (-0.00 - 0.00)
hematuria	0.16	4.49	**<.001**	0.16 (0.09 - 0.23)	0.03	0.54	0.599	0.03 (-0.09 - 0.16)
pyuria	0.03	1.39	0.174	0.03 (-0.01 - 0.08)	0.02	0.98	0.345	0.02 (-0.02 - 0.06)
urea	0.72	1.68	0.102	0.72 (-0.12 -1.55)	-0.02	-0.04	0.967	-0.02 (-0.96 – 0.92)
Scr	0.14	2.34	**0.026**	0.14 (0.02 - 0.25)	0.14	1.55	0.142	0.14 (-0.04 - 0.32)
eGFR	-0.04	-1.69	0.102	-0.04 (-0.09 - 0.01)	0.02	0.52	0.608	0.02 (-0.05 - 0.92)
dsDNA	0.01	1.70	0.101	0.01 (-0.00 - 0.01)	0.00	0.60	0.560	0.00 (-0.00 - 0.01)
C3	-7.4	-2.37	**0.025**	-7.40 (-13.52 - -1.27)	6.68	1.60	0.129	6.68 (-1.48 - 14.85)
CRP	-0.12	-1.64	0.111	-0.12 (-0.26 - 0.02)	-0.14	-1.75	0.100	-0.14 (-0.30 - 0.02)
25(OH)-VD_3_	-0.35	-3.59	**0.001**	-0.35 (-0.54 - -0.16)	0.15	0.90	0.384	0.15 (-0.17 - 0.46)

The LN patient data from the ELISA validation cohort (n=16).

bold values: P <0.05.

### The sensitivity and specificity of uAfamin in the diagnostic evaluation of LN

**Figure 2:**
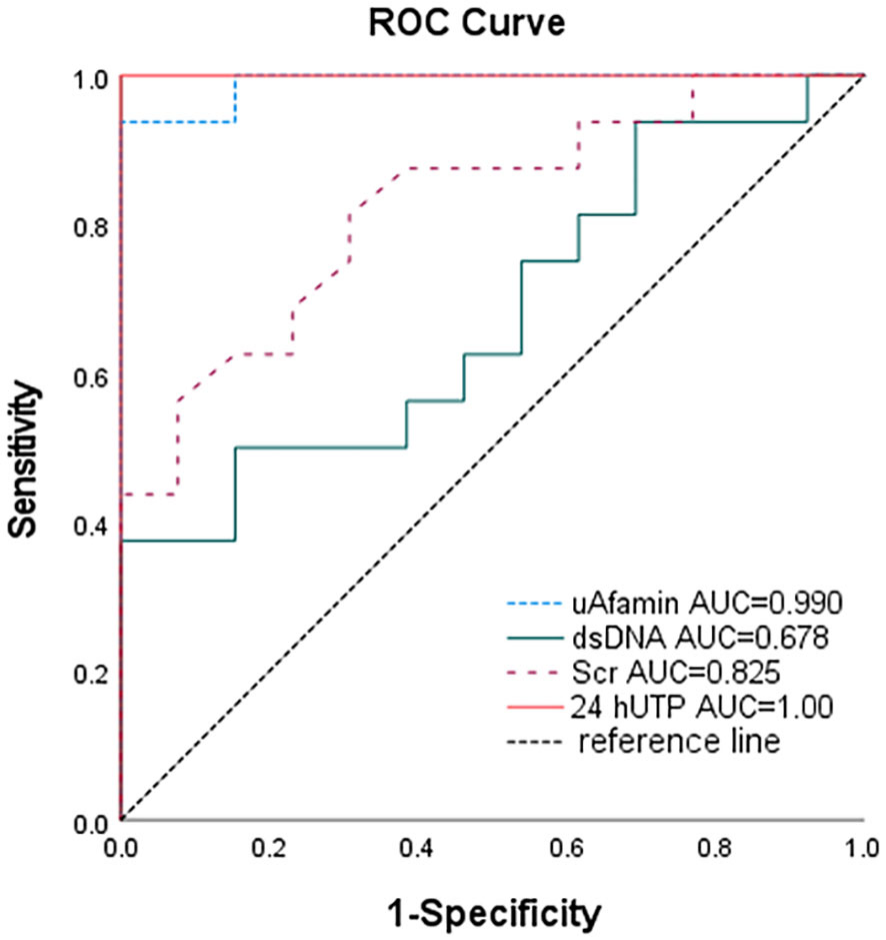
Sensitivity and specificity of uAfamin, dsDNA, Scr and 24hUTP between non-LN SLE (n=16) and LN-SLE (n=16). .

We constructed a nonparametric ROC curve to assess the utility of uAfamin in distinguishing SLE patients with LN. As shown in **Figure 2**, uAfamin exhibited excellent diagnostic performance, with an area under the curve (AUC) of 0.99 (*P* < 0.001), a sensitivity of 93.8%, and a specificity of 100%. This performance was superior to that of anti-dsDNA antibody and serum creatinine (Scr), and was second only to 24hUTP, which had an AUC of 1.0.

## Discussion

The identification of reliable clinical biomarkers for LN remains a critical unmet need in the management of SLE. Early diagnosis and timely intervention can significantly improve the long-term outcomes of SLE patients with renal involvement (4, 26). However, the often insidious onset and heterogeneous progression of LN complicate early detection and continuous monitoring. Although renal biopsy is the gold standard for diagnosing and classifying LN, its invasive nature precludes its repeated use for routine disease surveillance. Conventional serological markers, such as anti-dsDNA antibodies and complement levels (C3, C4), are widely used but lack sufficient sensitivity and specificity for accurately detecting renal activity (5, 27–29). In addition, traditional renal function parameters, such as urinalysis, Urine Protein-to-Creatinine Ratio (UPCR), 24hUTP, and serum creatinine, reflect damage relatively late in the inflammatory process. Hence, the discovery of non-invasive, sensitive biomarkers capable of forecasting LN and monitoring disease activity is of utmost importance.

In this study, TMT-based proteomic profiling of urine and plasma samples from HC, non-LN–SLE, and LN–SLE patients identified Afamin as a protein with markedly elevated levels in LN patients. This finding was subsequently validated by ELISA, which quantified significantly higher concentrations of both urinary and plasma Afamin in the LN–SLE group compared to the control groups. Our results align with the growing body of evidence that underscores Afamin as a promising biomarker for renal disease.

For instance, Afamin has been recognized as a urinary biomarker for diabetic nephropathy (14). Its relevance extends to other renal conditions: Sedic et al. reported elevated uAfamin in pediatric idiopathic nephrotic syndrome (18), while Zhao et al. observed a progressive increase in uAfamin levels in a rat model of focal segmental glomerulosclerosis, correlating with disease severity (15). In IgA nephropathy (IgAN), elevated uAfamin levels were detected in urinary proteomic analyses (17), and Kalantari et al. further proposed it as a predictive biomarker for IgAN severity (16). Additionally, Lu Pang et al. demonstrated that both uAfamin and the Afamin-to-creatinine ratio were elevated in primary membranous nephropathy and IgAN, showing positive correlations with urinary albumin and the albumin-to-creatinine ratio (19). Collectively, these findings underscore the consistent involvement of Afamin in diverse renal pathologies and support its candidacy as a biomarker in LN.

We hypothesized that Afamin could indicate both the presence and activity of LN in SLE patients. Our findings confirmed that Afamin levels were significantly elevated in patients with LN compared to those without renal involvement. Moreover, Afamin levels, particularly in urine, exhibited strong correlations with both renal (rSLEDAI) and systemic (SLEDAI-2K) disease activity scores, with the strongest association observed for rSLEDAI. This pattern suggests that elevated Afamin levels in SLE primarily reflect renal pathology. Notably, uAfamin demonstrated high diagnostic accuracy, comparable to 24 hUTP excretion. Collectively, these results indicate that uAfamin holds promise as a noninvasive biomarker for assessing renal activity, monitoring treatment response, and tracking disease progression in lupus patients.

**Figure 3:**
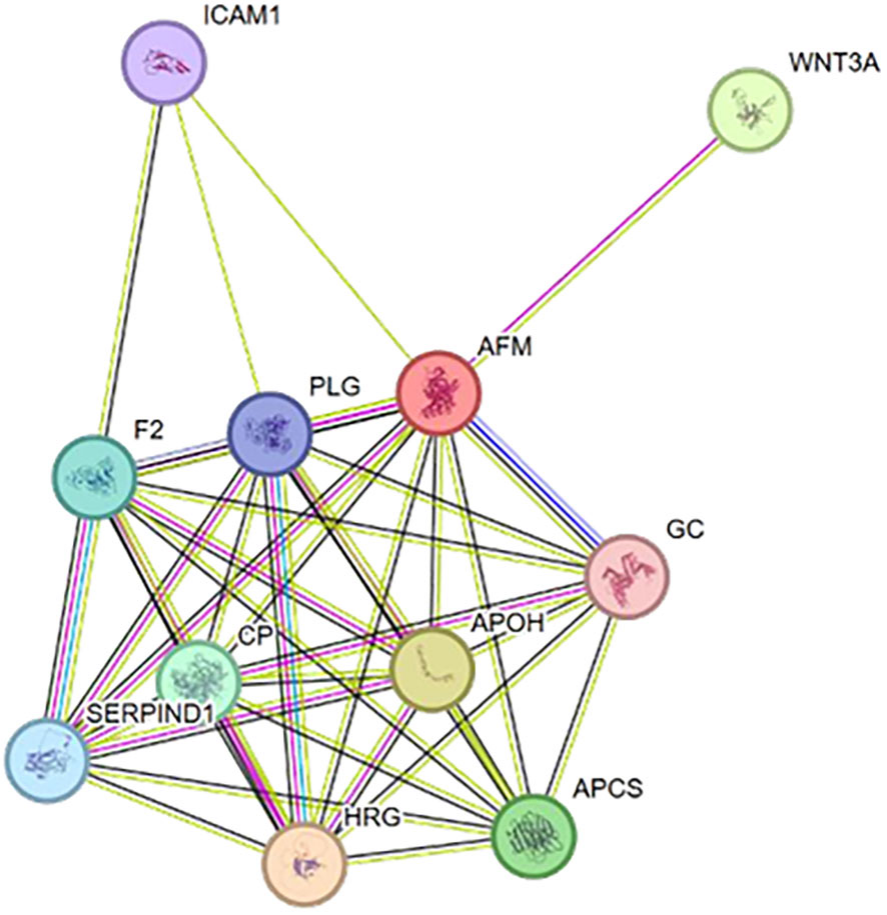
The interaction analysis of Afamin related protein by String.

Little is known about the mechanism by which Afamin contributes to the pathogenesis of LN. Although Kollerits et al. (30) found that higher serum afamin concentrations appear to be associated with a higher eGFR, less albuminuria, and a lower risk for future kidney failure in patients with CKD in a prospective cohort study with 6.5 years of follow-up. It contrasts with our observations. This difference may be explained by the distinct pathophysiology of LN (active inflammation) versus CKD (slower-progressing and non-inflammatory) and by racial differences in the cohorts. To elucidate potential mechanisms, we performed a STRING analysis, which revealed protein-protein interactions between Afamin and two key molecules: GC (31) (Vitamin D-binding protein) and Intercellular Cell Adhesion Molecule-1 (32) (ICAM-1) (**Figure 3**). GC plays a multifaceted role in immune and inflammatory responses. A study by Rupinder Kaur et al. (33) reported a negative correlation between serum Afamin and vitamin D levels, which aligns with our observation of significantly reduced serum 25(OH)-VD_3_ in LN patients, supporting this interaction. Consistent with this, we observed significantly lower serum 25(OH)-VD_3_ levels in LN patients compared to non-LN–SLE patients. Furthermore, 25(OH)- VD_3_ levels were significantly negatively correlated with both urinary and plasma Afamin levels, as well as with rSLEDAI in univariate linear regression analysis. Increasing evidence supports the immunomodulatory role of vitamin D (34, 35), which regulates the differentiation and activity of T and B lymphocytes and inhibits the production of autoantibodies (36). Reduced serum 25(OH)-VD_3_ levels have been associated with higher SLE disease activity and may contribute to the development of LN (37).

ICAM-1 is also intricately linked to LN progression (38–40). Active LN is histologically characterized by glomerular hypercellularity, immune complex deposition, necrosis, and inflammatory cell infiltration—the latter being a key feature distinguishing active from chronic lesions (26). As a critical vascular adhesion molecule, ICAM-1 mediates the recruitment of leukocytes from circulation into sites of renal inflammation and plays a fundamental role in immune cell signaling and activation. Previous studies have identified urinary soluble ICAM-1 as an early marker for detecting active LN. Building on these established roles and our protein interaction analysis, we hypothesize that Afamin may contribute to LN pathogenesis through functional interactions with GC and ICAM-1, although the precise nature and directionality of these relationships require further elucidation. Interestingly, we observed that uAfamin correlated more strongly with renal activity than its plasma counterpart, suggesting that urinary levels may more accurately reflect local renal involvement. While uAfamin is likely filtered through the glomerulus, its potential local production or secretion by renal tubular epithelial cells remains an open question for future research.

Histidine-rich glycoprotein (HRG) is a plasma glycoprotein known to bind heparan sulfate on endothelial cells in various tissues, including the kidney. It functions as an adapter protein and has been implicated in processes such as immune complex clearance, pathogen removal, and cell chemotaxis. We speculate that HRG may play a restorative role in renal tissue, possibly contributing to the modulation of Afamin levels in LN. Further investigation is needed to elucidate the potential interplay between HRG and Afamin in the context of LN.

Several limitations should be considered in this study. First, the sample size was relatively small, particularly within LN subgroups, which may affect the statistical power and generalizability of our findings. Second, the influence of LN pathological classification on Afamin expression was not evaluated; thus, variations across different histological subtypes remain unclear. Additionally, while patients with pre-existing hypertension were excluded, those who developed hypertension after SLE onset were not analyzed separately, which may influence the specificity of uAfamin as a biomarker for LN. Finally, Afamin levels in LN patients before and after treatment have not yet been assessed. In subsequent follow-up studies, we intend to monitor this dynamic parameter to evaluate its utility in diagnosing treatment response and disease recurrence.

In summary, our findings indicate that Afamin is significantly elevated in both urine and plasma of patients with LN, with urinary Afamin demonstrating a strong correlation with renal disease activity. These results support the potential of uAfamin as a non-invasive biomarker for detecting LN and monitoring renal involvement in SLE. Future studies with larger and more diverse cohorts are warranted to validate its diagnostic and prognostic value across LN subtypes and to further explore its role in the pathogenesis of LN.

## Data Availability

The original contributions presented in the study are included in the article/**Supplementary Material**, further inquiries can be directed to the corresponding authors.
