# Supplementary material for: Urine Afamin as a biomarker of lupus nephritis
Source: Front Immunol. 2025 Nov 28;16:1696288. doi: 10.3389/fimmu.2025.1696288 (PMC12698515; doi:10.3389/fimmu.2025.1696288)
Supplement: Supplementary file 1 [file Supplementaryfile1.doc]

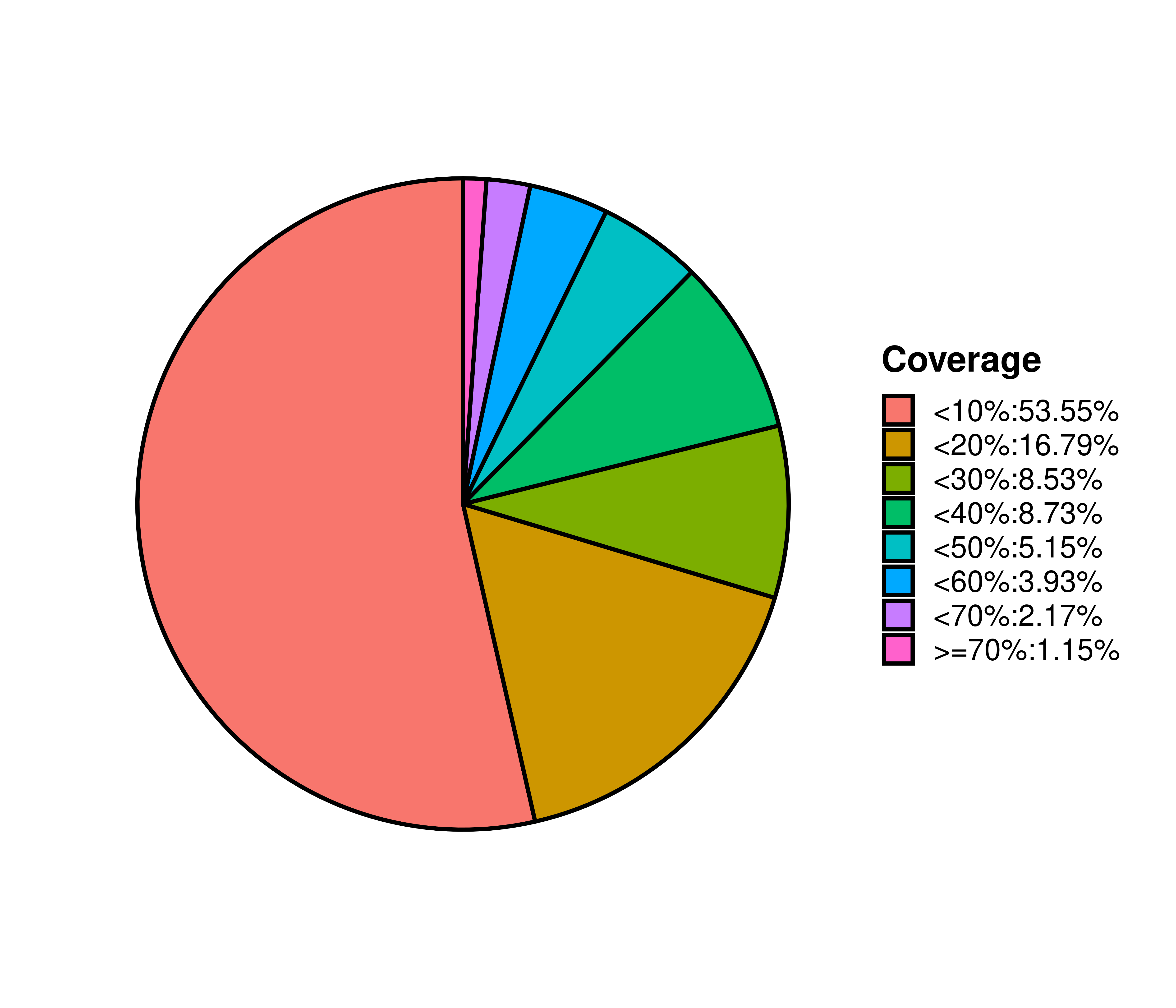


**
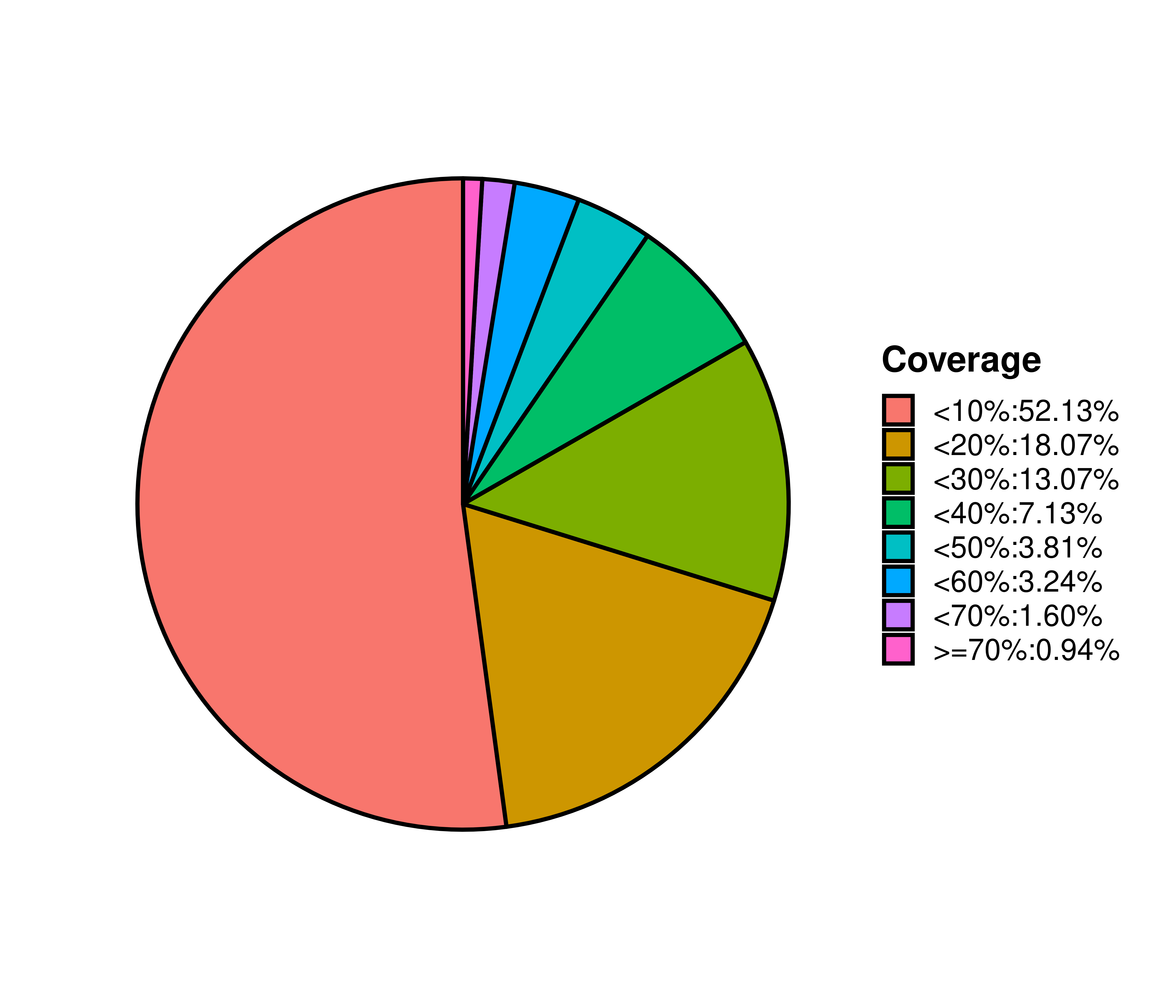
**

**E**

**F**

**G**

**H**

Figure1 TMT showed the levels of protein abundance of change from urinary and plasma.  The differentially distribution of protein about polypeptide identification numbers, protein molecular weight, polypeptide length and protein identification peptide coverage displayed in A-D (proteins from urinary) and E-G (proteins from plasma)

| **Supplementary Table 1** Clinical and laboratory features of the patients of proteomics | | | | |
| --- | --- | --- | --- | --- |
|  | LN-SLE | Non-LN SLE | statistic | P value |
|  | n=15 | n=15 |  |  |
| age(year) | 37.02±9.85 | 39.46±8.31 | t=-0.73 | 0.324 |
| male/female, n (%) | 3(20.00)/12(80.00) | 5(33.33)/10(66.67) | - | 0.589 |
| SLE disease duration(year) | 3.8(1.0,10.9) | 3.6(1.2,9.7) | z=-0.18 | 0.873 |

LN lupus nephritis, SLE systemic lupus erythematosus
